# Electronic evidence of temperature-induced Lifshitz transition and topological nature in ZrTe_5_

**DOI:** 10.1038/ncomms15512

**Published:** 2017-05-23

**Authors:** Yan Zhang, Chenlu Wang, Li Yu, Guodong Liu, Aiji Liang, Jianwei Huang, Simin Nie, Xuan Sun, Yuxiao Zhang, Bing Shen, Jing Liu, Hongming Weng, Lingxiao Zhao, Genfu Chen, Xiaowen Jia, Cheng Hu, Ying Ding, Wenjuan Zhao, Qiang Gao, Cong Li, Shaolong He, Lin Zhao, Fengfeng Zhang, Shenjin Zhang, Feng Yang, Zhimin Wang, Qinjun Peng, Xi Dai, Zhong Fang, Zuyan Xu, Chuangtian Chen, X. J. Zhou

**Affiliations:** 1 Beijing National Laboratory for Condensed Matter Physics, Institute of Physics, Chinese Academy of Sciences, Beijing 100190, China; 2 University of Chinese Academy of Sciences, Beijing 100049, China; 3 Collaborative Innovation Center of Quantum Matter, Beijing 100871, China; 4 Military Transportation University, Tianjin 300161, China; 5 Technical Institute of Physics and Chemistry, Chinese Academy of Sciences, Beijing 100190, China

## Abstract

The topological materials have attracted much attention for their unique electronic structure and peculiar physical properties. ZrTe_5_ has host a long-standing puzzle on its anomalous transport properties manifested by its unusual resistivity peak and the reversal of the charge carrier type. It is also predicted that single-layer ZrTe_5_ is a two-dimensional topological insulator and there is possibly a topological phase transition in bulk ZrTe_5_. Here we report high-resolution laser-based angle-resolved photoemission measurements on the electronic structure and its detailed temperature evolution of ZrTe_5_. Our results provide direct electronic evidence on the temperature-induced Lifshitz transition, which gives a natural understanding on underlying origin of the resistivity anomaly in ZrTe_5_. In addition, we observe one-dimensional-like electronic features from the edges of the cracked ZrTe_5_ samples. Our observations indicate that ZrTe_5_ is a weak topological insulator and it exhibits a tendency to become a strong topological insulator when the layer distance is reduced.

## Abstract (web summary)

To understand the anomalous electronic transport properties of ZrTe_5_ remains an elusive puzzle. Here, Zhang *et al*. report direct electronic evidence to the origin of the resistivity anomaly and temperature induced Lifshitz transition in ZrTe_5_, indicating it being a weak topological insulator.

The transition metal pentatellurides like ZrTe_5_ and HfTe_5_ (ref. 1) have attracted considerable interest since the last 70s because they exhibit unusual transport properties characterized by a strong resistivity peak^2^,^3^ accompanied by a sign reversal of the Hall coefficient and thermopower across the peak temperature^4^,^5^,^6^. The origin of such transport property anomalies has been a subject of a long-time debate but remains unclear, with explored possibilities of structural phase transition^3^,^7^, formation of a charge/spin density wave^8^, polaronic behaviour^9^, a semimetal–semiconductor transition^6^ or temperature-induced band shift^10^. With the emergence of topological materials, including topological insulators^11^,^12^,^13^, three-dimensional Dirac semimetals^14^,^15^,^16^,^17^,^18^,^19^,^20^,^21^,^22^,^23^ and three-dimensional Weyl semimetals^24^,^25^,^26^,^27^,^28^,^29^,^30^,^31^,^32^,^33^, ZrTe_5_ and HfTe_5_ have ignited renewed interest as a candidate of a novel topological material. While many three-dimensional topological insulators have been predicted and discovered, two-dimensional topological insulators, also known as quantum spin Hall insulators that can support topologically protected helical edge modes inside a bulk insulating gap and lead to dissipationless transport, are rare^34^,^35^,^36^, especially in natural compounds^13^. It was predicted that single-layer ZrTe_5_ is a two-dimensional topological insulator with a large bulk bandgap, while bulk ZrTe_5_ may host a possibility of realizing a temperature-driven topological phase transition between the weak and strong topological insulators^37^. However, a number of recent experiments on ZrTe_5_ point to its being a three-dimensional Dirac semimetal^38^,^39^,^40^ or a quasi-two-dimensional Dirac semimetal^41^. Added to the interest of ZrTe_5_ is the recent observation of superconductivity under high pressure^42^. Direct investigation on the electronic structure of ZrTe_5_ is highly desired in understanding the electronic origin of the transport property anomaly, and in uncovering the exact nature of the topological state in ZrTe_5_. Angle-resolved photoemission spectroscopy (ARPES)^43^ can provide direct information on the electronic structure of materials in addressing the above-mentioned prominent issues in ZrTe_5_. However, high-resolution comprehensive ARPES measurements on ZrTe_5_, especially the low-energy electronic structure, are still lacking^6^,^10^,^38^.

In this paper, we present detailed ARPES measurements on ZrTe_5_ with unprecedented energy and momentum resolutions, by taking advantage of our latest-generation laser-based ARPES system, which can cover two-dimensional momentum space simultaneously. For the first time, we find that the electronic property of ZrTe_5_ is dominated by two branches of bands with nearly linear dispersion, a valence band and a conduction band, at the Brillouin zone centre. There is an energy gap that separates these two branches of bands; the gap decreases with decreasing temperature but persists to a very low temperature (∼2 K). The overall electronic structure exhibits a marked temperature dependence that shifts down with decreasing temperature. This results in an evolution from a *p*-type semimetal with a hole-like Fermi pocket at high temperature above 135 K, to a semiconductor around 135 K where its resistivity shows a peak, to an *n*-type semimetal with an electron-like Fermi pocket at low temperature. These results constitute direct electronic evidence of a temperature-induced Lifshitz transition in ZrTe_5_. They provide a natural understanding on the underlying origin of the resistivity anomaly at ∼135 K and its sign reversal of Hall coefficient and thermopower across the same temperature. We also observe one-dimensional-like electronic features associated with the edges of some cracked ZrTe_5_ samples. Our observations indicate that ZrTe_5_ is a weak topological insulator that shows a tendency to become a strong topological insulator when the layer distance is reduced.

## Results

### Sample characterization

**Figure 1:**
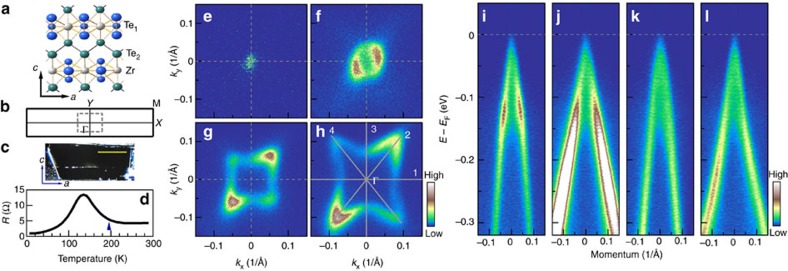
Fermi surface and band structure of ZrTe_5_ measured at 195 K. (**a**) Top view of the bulk crystal structure (*ac* plane) of the ZrTe_5_ sheet. The blue and green spheres represent Te atoms and the grey ones represent Zr atoms. ZrTe_5_ crystal is constructed from stacking of the ZrTe_5_ sheets along the *b* axis (perpendicular to the *ac* plane). (**b**) Surface Brillouin zone corresponding to *ac* plane. High-symmetry points are indicated. The central dashed-line square indicates the measured momentum space covered by our ARPES mapping in **e**–**h**. (**c**) The cleaved surface morphology of a thick ZrTe_5_ sample, which is flat and mirror-like. The scale bar in this panel represents 1 mm. (**d**) Temperature dependence of resistivity for our ZrTe_5_ single-crystal samples; there is a clear resistivity peak at ∼135 K. (**e**–**h**) Constant energy contours of ZrTe_5_ at different binding energies of 0, 100, 200 and 300 meV, respectively. The spectral intensity is integrated within 10 meV with respect to each binding energy. The measurement geometry is set under **s** polarization. (**i**–**l**) Band structures measured along typical cuts 1, 2, 3 and 4, respectively. The location of the momentum cuts is shown in **h** by thick grey lines.

**Figure 2:**
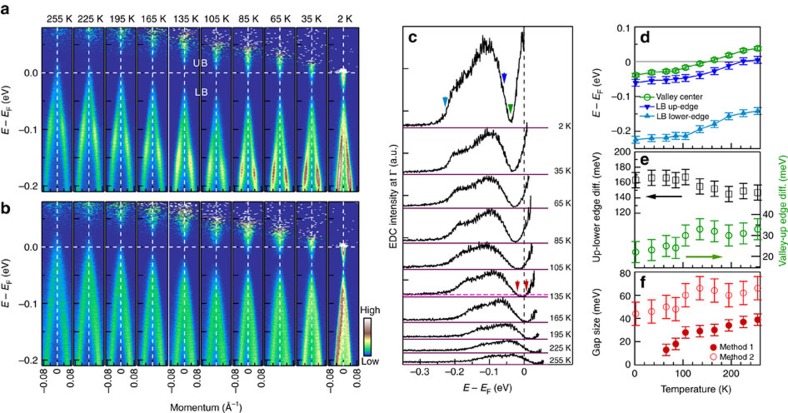
Temperature evolution of the band structures in ZrTe_5_. (**a**,**b**) Temperature-dependent band structures measured along Γ–*X* (cut 1 in Fig. 1h) and Γ–*Y* directions (cut 3 in Fig. 1h). The corresponding Fermi distribution functions are divided out to reveal features above the Fermi level. (**c**) EDCs at Γ point at different temperatures. For clarity, the EDCs are offset along vertical axis, with zero intensity represented by the horizontal purple lines. The EDCs consist of the LB valence band and the UB conduction band, with a valley separating in between them with its centre marked by a green triangle as for the 2 K EDC. The LB band is encompassed by a lower-binding-energy edge (marked by a deep blue arrow as for the 2 K EDC) and high-binding-energy edge (marked by a light blue arrow as for the 2 K EDC). (**d**) The energy positions of the high-binding-energy edge (light blue triangles) and lower-binding-energy edge (deep blue triangles) of the LB band, together with the centre of the valley bottom (green empty circles) at different temperatures. (**e**) Energy difference between the two edges of the LB band (black empty squares) and between the lower-binding-energy edge of the LB band and the centre of the valley bottom (green empty circles) at different temperatures. (**f**) Energy gap size at different temperatures estimated from two methods. In method 1, we take the low-intensity region of EDCs at high binding energy 0.30–0.35 eV as the background (horizontal dashed pink line for 135 K EDC) that intersects with the valley bottom at two points (as marked by two red arrows for 135 K EDC). The gap size (solid red circles) is estimated from the distance between these two points. In method 2, the gap size (empty red circles) is estimated as twice the energy difference between the valley bottom centre and the upper edge of the LB band. The error bar in **d**–**f** is based on the uncertainty in determining the energy position of the edges and valley centre in EDCs.

**Figure 3:**
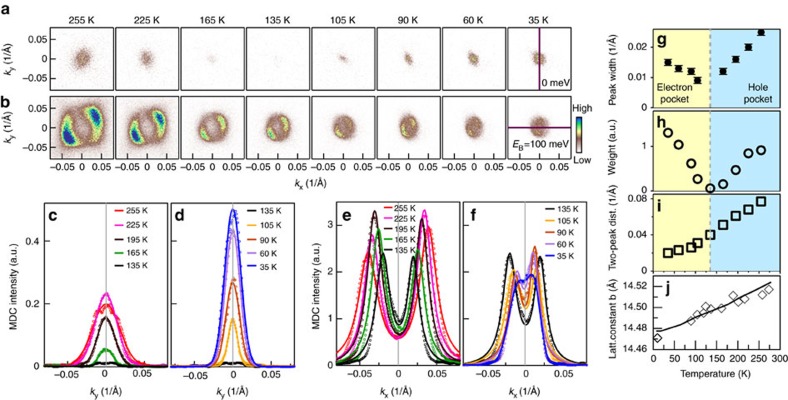
Temperature-induced Lifshitz transition in ZrTe_5_. (**a**) Fermi surface evolution with temperature when ZrTe_5_ is cooled down from 255 to 35 K. (**b**) Corresponding constant energy contour evolution with temperature at a binding energy of 100 meV. (**c**,**d**) Momentum distribution curves (MDCs) at the Fermi level (*E*_F_) measured along the vertical momentum cut (Γ–*Y* direction) as indicated in the 35 K panel of **a** at different temperatures (open circles). To improve data statistics, the MDCs are obtained by integrating within ±5 meV energy window with respect to the Fermi level. Because multiple-peak features are not resolved, we fitted the MDCs by a Gaussian to estimate the pocket size and signal intensity. The fitted MDC width and spectral weight are shown in **g**,**h**, respectively. (**e**,**f**) MDCs at a binding energy of 100 meV measured along the horizontal momentum cut (Γ–*X* direction) as indicated in the 35 K panel of **b** at different temperatures (open circles). The MDCs are obtained by integrating within ±5 meV energy window at the binding energy of 100 meV. Here the MDCs show two clear peaks that are approximated by two Lorentzians or Gaussians. The distance between the two peaks is shown in **i** that is related to the area of the constant energy contours in **b**. (**g**) Temperature dependence of the MDC width (full-width at half-maximum) extracted from **c**,**d**. (**h**) Temperature dependence of the MDC weight, the integrated area of MDCs, extracted from **c**,**d**. (**i**) Temperature dependence of the two-peak distance of MDCs in **e**,**f**. (**j**) Temperature dependence of the lattice constant *b* with the measured data (black diamonds) replotted from ref. 62 and the fitted line (black line). The lattice constant *b* is related to the interlayer spacing.

ZrTe_5_ is a layered compound that crystallizes in the orthorhombic crystal structure^1^. It is constructed from layer-stacking of the ZrTe_5_ sheets along the *b* axis. The two-dimensional ZrTe_5_ sheet (Fig. 1a) features a trigonal prismatic ZrTe_6_ chains running along the *a* axis that are linked together along the *c* axis via zigzag chains of Te atoms. Each ZrTe_5_ sheet is nominally charge neutral, and the distance between the adjacent two ZrTe_5_ sheets along the *b* axis is quite large (∼7.25 Å), giving rise to a weak interlayer coupling^37^. Figure 1b shows the projected surface Brillouin zone for ZrTe_5_ with the high symmetry momentum points labelled in a standard way. Figure 1c shows the morphology of a cleaved ZrTe_5_ surface that is smooth and mirror-like; the data presented in Figs 1, 2, 3 were taken on this kind of surface. The resistivity-temperature data of our ZrTe_5_ sample (Fig. 1d) exhibits a prominent peak at ∼135 K. This is consistent with the typical ZrTe_5_ samples reported before^2^,^3^,^4^,^6^,^7^,^8^,^9^,^10^,^41^,^42^,^44^,^45^ (see Methods for the sample details).

### Fermi surface and band structure

The ARPES data are taken with our new laser-based system equipped with the latest-generation time-of-flight analyser. It not only has high energy and momentum resolutions but also has a new capability of covering two-dimensional momentum space simultaneously (see Methods for experimental details). Figure 1e–h shows the constant energy contours of ZrTe_5_ measured at 195 K, which represent the spectral intensity distribution at the Fermi level (Fig. 1e) and at a few binding energies of 100 (Fig. 1f), 200 (Fig. 1g) and 300 meV (Fig. 1h). The corresponding band structures are shown in Fig. 1i–l measured along several typical momentum cuts as indicated in Fig. 1h. We have checked the effect of the polarization geometry on the measured results. The measured constant energy contours and band structures are similar under two typical s and p polarization geometries, although the spectral weight distribution in the momentum space shows a clear polarization dependence (Supplementary Fig. 1).

**Figure 4:**
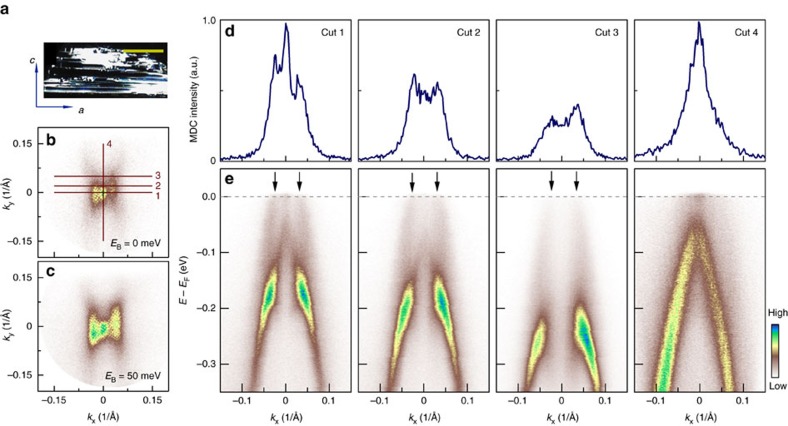
Observation of weak topological insulator feature in some ZrTe_5_ samples. (**a**) Cleaved surface morphology of some thin ZrTe_5_ samples or thin ZrTe_5_ samples after temperature cycling. There are one-dimensional thread-like structures running along the *a* axis. The additional features presented here are taken on this kind of samples. The scale bar in this panel represents 1 mm. (**b**,**c**) Constant energy contours of ZrTe_5_ measured around 15 K at binding energy of 0 and 50 meV, respectively. Besides the tiny pocket near the Γ point, there are two nearly one-dimensional spectral streaks on both sides of Γ point running along Γ–*Y* direction. Band structures measured along momentum cuts 1, 2, 3 and 4 are shown in **e**. The locations of these momentum cuts are indicated in **b**. Corresponding MDCs at Fermi level for band structures in **e** are shown in **d**. In addition to the usual bands observed in ZrTe_5_ as shown in Fig. 1i–l, addition bands are observed here that are marked by the arrows in **e**.

The constant energy contour at the Fermi level shows a tiny spot at the Brillouin zone centre, Γ point (Fig. 1e). With increasing binding energy, it grows in area and evolves into a warped rectangle at high binding energies of 200 (Fig. 1g) and 300 meV (Fig. 1h). This is consistent with the observation of hole-like bands below the Fermi level as seen in Fig. 1i–l. These hole-like bands show nearly linear dispersions over a wide energy range. They also exhibit a strong anisotropy: the bands are steeper along the horizontal or vertical high-symmetry directions (Fig. 1i,k for cuts 1 and 3 in Fig. 1h) with a Fermi velocity of ∼4 eV·Å (corresponding to 6.3 × 10^5^ m s^−1^, 1/480 *c* with *c* being the speed of light) than those along the momentum cuts 2 (Fig. 1j) and 4 (Fig. 1l) with a Fermi velocity of ∼2.3 eV·Å (corresponding to 3.6 × 10^5^ m s^−1^, 1/830 *c*). Detailed lineshape analysis of these hole-like bands at Γ indicates that they are broad; the corresponding photoemission spectra (energy distribution curves, EDCs) are not composed of a single sharp peak or two sharp peaks, but represent a spectral continuum encompassed by two edges (Supplementary Fig. 2).

To understand the measured electronic structure, we have performed band structure calculations of ZrTe_5_ (see Methods for details of the band structure calculations). The calculated band structures (Supplementary Figs 3 and 4) and constant energy contours (Supplementary Fig. 5) are basically consistent with the previous reports^37^,^46^. The evolution of the constant energy contours near Γ point with the increasing binding energy (Fig. 1e–h) from a spot to a small circle to a warped rectangle is in good agreement with the calculated results (Supplementary Fig. 5), considering a chemical potential difference in the comparison. Band structure calculations on bulk ZrTe_5_ give only one sharp hole-like band near Γ in this case that is apparently not consistent with our measurements. The slab calculations (Supplementary Fig. 4b), on the other hand, provide a good explanation on this behaviour. This broad band feature can be understood reasonably well by the finite *k*_*z*_ effect, consistent with the results of the slab band calculations (Supplementary Fig. 4b,d).

### Temperature evolution of the electronic structures

We have carried out detailed ARPES measurements on ZrTe_5_ at different temperatures to understand the origin of the anomalous transport properties and examine on possible temperature-induced topological phase transition. Figure 2 shows the temperature dependence of the energy bands measured along two high-symmetry momentum cuts. The corresponding temperature dependence of the constant energy contours is shown in Fig. 3. These results are highly reproducible by measuring on the same sample with cycles of cooling down and warming up (Supplementary Fig. 6), by cleaving samples at high and low temperatures, and by measuring on many different samples. The band images (Fig. 2a,b) are obtained by dividing the original data with their corresponding Fermi distribution functions, making it possible to reveal features above the Fermi level at relatively high temperatures.

From Fig. 2a,b, it is clear that the band structure near Γ consists of two branches, one is the upper branch (UB) at lower binding energy or above the Fermi level that corresponds to the electron-like conduction band, while the other is the lower branch (LB) at high binding energy that corresponds to the hole-like valence band. In between the UB and LB bands, there is a region with strongly suppressed spectral weight that we call it valley region. The bands at Γ point show a strong temperature dependence; the overall band structure shifts down to high binding energy with decreasing temperature. Specifically, the LB band touches the Fermi level at high temperature (255 K in Fig. 2a,b); it shifts down with decreasing temperature and sinks well below the Fermi level at low temperatures. On the other hand, the UB band is well above the Fermi level at high temperature (255 K in Fig. 2a,b), moves down with decreasing temperature and crosses the Fermi level at very low temperature (for example, 35 and 2 K in Fig. 2a,b). Such a band shift with temperature is consistent with those reported before^6^,^10^.

Although the overall band structure shifts down with decreasing temperature, we find that it is not a rigid band shift. To keep track on the temperature evolution of the bands in a more quantitative way, we plot the photoemission spectra (EDCs) at Γ point measured at different temperatures in Fig. 2c. The EDCs consist of signals from the LB band and the UB band, with a valley separating in between them. The LB band is visible in the entire measured temperature range, which is encompassed by a lower-binding-energy upper edge and high-binding-energy lower edge. The UB band is invisible above *E*_F_ at high temperatures (255 K), moves down with decreasing temperature and part of it becomes visible at low temperatures. Figure 2d shows the energy positions of the upper edge of the LB band, lower edge of the LB band and the centre of the valley bottom. The two edges of the LB band and the valley centre show a similar trend of temperature dependence; the overall energy shift from 255 to 2 K is on the order of ∼70 meV. A close examination indicates that the LB band gets wider with decreasing temperature as seen from the energy difference between the upper and lower edges of the LB band (Fig. 2e). On the other hand, the U-shaped valley becomes steeper with decreasing temperature, seen from the energy difference between the upper edge of the LB band and the valley centre (Fig. 2e). These indicate that the band shift with temperature is not a strictly rigid band shift. We notice that 135 K is a characteristic temperature where the valley centre crosses the Fermi level (Fig. 2c,d). We also note that the energy shift of bands near Γ with temperature is faster in the high-temperature region of 165–255 K than that in the lower-temperature 2–105 K region (Fig. 2d).

The strong suppression of the spectral weight in the valley region is indicative of an gap opening between the LB band and the UB band (Fig. 2a,b). As seen in Fig. 2c, at high temperatures, the valley bottom in EDCs shows a flat region with an intensity close to zero when taking the signal background into account. This suggests a true gap opening at high temperatures. We estimate the gap size in two ways. In the first method, we choose the high-binding-energy region at 0.30–0.35 eV in EDCs, which is very weak and slightly above zero intensity as the background line. The line intersects with the valley bottom at two points and the distance between these two points are taken as an estimation of the gap size. Compared with zero intensity background, this method slightly overestimates the gap size, but it provides an objective and direct way in extracting the gap value and the difference is small compared to that obtained from zero intensity background. The energy gap obtained in this way is large at high temperatures (∼40 meV for 255 K), and gets smaller with decreasing temperature (Fig. 2f). At very low temperatures, such as 35 and 2 K, the spectral weight near the valley bottom increases that is above the zero intensity. In this case, there is no more true gap opening so we cannot determine the gap size using the same criterion as used at high temperatures. However, we believe there remains a gap opening near the valley region because the spectral weight is still strongly suppressed, a situation similar to the pseudogap observed in high-temperature cuprate superconductors^43^. It is also possible that besides a true gap opening there is a new in-gap state produced by other mechanism that fills in the gap region and grows with decreasing temperature. If there is no gap opening and a Dirac cone-like structure is formed, one would expect to see a peak at the Dirac point that is not consistent with the present result. This leads us to take another way to estimate the gap size. If we assume a Dirac cone structure as a reference point that has zero gap, the gap opening would split the LB and the UB bands, and causes a spectral weight suppression at the gapped valley region. In this case, the distance between the LB and the UB bands can be used as a measure of the gap size. Assuming the LB and the UB bands show similar temperature-dependent splitting from the valley centre, the gap size can be estimated as twice the distance between the position of the upper edge of the LB band and the position of the valley centre, as shown in Fig. 2f (red empty circles). In this case, the gap size still decreases with decreasing temperature, but it persists in the entire temperature range we have measured.

### Temperature-induced Lifshitz transition

The temperature dependence of the Fermi surface provides a clear evidence of a temperature-induced Lifshitz transition in ZrTe_5_. Figure 3 shows the temperature evolution of the constant energy contours at the Fermi level (Fig. 3a) and at a binding energy of 100 meV (Fig. 3b) for ZrTe_5_. The corresponding momentum distribution curves across the Γ point at different temperatures are shown in Fig. 3c–f for the two cases, which provide information on the temperature evolution of the pocket size (Fig. 3g) and the spectral weight at the Fermi level (Fig. 3h). At a high temperature like 255 K, there is a tiny hole-like pocket at Γ (left-most panel in Fig. 3a), consistent with a hole-like band crossing the Fermi level in Fig. 2a,b. With decreasing temperature, the pocket near Γ shrinks in size, becomes invisible at 135 K, then emerges again below 135 K. Its size increases with further decreasing of temperature and it becomes an electron-like pocket as seen from bands in Fig. 2a,b. Therefore, there is a clear Lifshitz transition that occurs across ∼135 K where the Fermi surface topology transforms from a hole-like pocket at high temperature to an electron-like pocket at low temperature. It is interesting to note that a temperature-induced Lifshitz transition is also reported in another transition metal dichalcogenide WTe_2_ (ref. 47). For the constant energy contours at a binding energy of 100 meV (Fig. 3b), the pocket size at the Γ point keeps shrinking with decreasing temperature, as seen from the distance of two peaks in momentum distribution curves (Fig. 3e,f,i). As seen in Fig. 2, the band at 100 meV binding energy represents solely the LB valence band over the entire temperature range we measured. It is natural that the pocket size shrinks when the hole-like band moves to high binding energy with decreasing temperature. In addition to the bands at Γ point, we also observe another band (denoted as *β* band) that crosses the Fermi level at low temperature and forms four electron pockets at Brillouin zone boundary when we scan the entire momentum space of the first Brillouin zone (Supplementary Fig. 7). This is consistent with the band structure calculations (Supplementary Fig. 5d). Together with the Γ point bands, the *β* band also shifts down when temperature decreases (Supplementary Fig. 8). Except for the near Γ point bands and *β* band described above, no other band is detected over the entire first Brillioun zone in the temperature range we measured (Supplementary Fig. 7).

## Discussion

The identification of a temperature-induced Lifshitz transition provides a natural explanation on the origin of the transport property anomalies in ZrTe_5_. Because there is an energy gap between the LB band and the UB band, and both bands shift with temperature (Fig. 2), it is clear that at high temperature, when the Fermi surface is a hole-like pocket, it is a *p*-type semimetal. With decreasing temperature, when the hole-like valence band sinks down to below the Fermi level and the Fermi level lies in the gapped region, ZrTe_5_ enters into a semiconducting state. At ∼135 K, the Fermi level lies close to the centre of the energy gap. With further decreasing of temperature, the UB conduction band moves downwards to touch the Fermi level. The Fermi surface becomes an electron-like pocket and ZrTe_5_ changes into an *n*-type semimetal at low temperature. Therefore, ZrTe_5_ undergoes a transition from a *p*-type semimetal at high temperature, to a semiconductor in a narrow temperature region around 135 K, to an *n*-type semimetal at low temperature. The temperature evolution of the spectral weight at the Fermi level (Fig. 3h) is consistent with this picture. We note that around 135 K the transport properties of ZrTe_5_ are mainly dictated by the bands near Γ point; the other *β* band does not cross the Fermi level until very low temperature that will add more electrons into ZrTe_5_ and further reduce its resistivity (Supplementary Figs 8 and 9). This provides a natural explanation on the resistivity maximum at ∼135 K in the transport measurement. It also accounts for the sign reversal of the Hall coefficient and thermopower^4^,^6^ because it corresponds to charge carrier change from *p*-type at high temperature to *n*-type at low temperature across ∼135 K.

Our present results pose a new issue on the origin of the chemical potential variation with temperature in ZrTe_5_, that is, how temperature can induce a marked chemical potential shift in ZrTe_5_ without obvious charge carrier doping. In terms of usual understanding, our temperature-dependent results indicate that extra holes are present above ∼135 K, no carriers present in the semiconducting state around 135 K and extra electrons appear below ∼135 K. Without external doping mechanism, these seem to violate the charge balance in the temperature variation process. We believe the measured temperature-dependent behaviours represent intrinsic properties of ZrTe_5_, not artefact caused by extrinsic factors like trivial absorption/desorption process. First, the measurements were performed in ultrahigh vacuum and in different ARPES systems; the results are reproducible. Second, the results are consistent with other ARPES results on similar ZrTe_5_ sample with similar resistivity anomaly temperature^6^,^10^. Third, our ARPES measurements identified a Lifshitz transition at ∼135 K that corresponds to the resistivity anomaly temperature; it is hard to believe this is coincidental. Fourth, most importantly, our ARPES results are consistent with the bulk Hall coefficient and thermopower measurements that indicate a charge carrier change from hole-like at high temperature to electron-like at low temperature across ∼135 K (refs 4, 5, 6). Such an unusual temperature-dependent chemical potential shift was considered to be a characteristic of an intrinsic semiconductor^6^. It may also be related to charge carrier localization/delocalization on changing temperature. It is well known that the chemical potential can exhibit a significant shift with temperature in a degenerate semiconductor^48^. It is possible that the ZrTe_5_ samples we measured contain defects or impurities; whether ZrTe_5_ really behaves like a degenerate semiconductor needs to be further explored. Considering similar temperature dependence of the bandgap (Fig. 2f) and the lattice parameter *b* (Fig. 3j), it is also interesting to investigate whether the variation of the interlayer interaction with temperature might give rise to such an unusual electronic state transition. While we do not have a clear answer on the origin yet, the marked charge carrier change with temperature may hide some deep physical mechanism that calls for further investigations.

Our detailed electronic structure measurements of ZrTe_5_ provide direct information to examine on the nature of its possible topological state, that is, whether it is a topological insulator^37^ or a quasi-two-dimensional^41^ or three-dimensional Dirac semimetal^38^,^39^,^40^. If ZrTe_5_ is a quasi-two-dimensional semimetal, one would observe a Dirac cone in the *ac* plane that we have measured. The absence of such a Dirac cone in our measurements clearly rules out such a scenario. For a similar reason, the gap opening between the LB valence band and the UB conduction band, especially its variation with temperature and its persistence down to the lowest temperature we measured (Fig. 2c,f) are not compatible with the three-dimensional Dirac semimetal picture for ZrTe_5_. Our electronic structure measurements are in good agreement with the transport measurements that show a resistivity anomaly and a reversal between electron- and hole-like charge carriers. One may argue whether the gap opening we observed is due to *k*_*z*_ effect because, in principle, the three-dimensional Dirac cone can only be seen at particular *k*_*z*_ values. Our measurements using different photon energies indicate that *k*_*z*_ for the 6.994 eV laser is close to the basal *k*_*z*_=0 plane (Supplementary Fig. 10). In particular, the gradual temperature-dependent change of the relative position between the UB and the LB bands near Γ point provides strong evidence on the gap opening, irrespective of *k*_*z*_ location, which is not consistent with the three-dimensional Dirac cone picture for ZrTe_5_. We note that the inconsistency on the nature of topological state in ZrTe_5_ may be due to sample difference. The ZrTe_5_ we measured here has a resistivity anomaly at ∼135 K that is consistent with most of the samples reported before^2^,^3^,^4^,^6^,^7^,^8^,^9^,^10^,^41^,^42^,^44^, ^45^. On the other hand, for the reports of three-dimensional Dirac semimetal^38^,^39^,^40^, the ZrTe_5_ samples were grown by a different method and exhibit a resistivity anomaly at a much lower temperature, ∼60 K. While our ARPES results are consistent with previous reports^6^,^10^ on the ZrTe_5_ samples with similar resistivity anomaly temperature ∼135 K, they are quite different from those on the ZrTe_5_ sample with a ∼60 K resistivity anomaly^38^.

Band structure calculations predict that single-layer ZrTe_5_ is a two-dimensional topological insulator^37^. Our ARPES results in Figs 1, 2, 3 are hard to provide a definitive answer on this issue because the ZrTe_5_ sample we measured is bulk instead of a single-layer form. However, some additional quasi-one-dimensional features we observed in some cleaved thin samples appear compatible with the edge states in ZrTe_5_. For some cleaved thin ZrTe_5_ samples, there may appear some one-dimensional line structures on the surface (Fig. 4a); the population of these structures can be further increased by deliberately cycling the sample temperature up and down many times. In these samples, we can observe additional bands growing on top of the original bulk bands (Fig. 4e). These extra bands form quasi-one-dimensional intensity streaks running along the *k*_*y*_ direction, which is perpendicular to the one-dimensional ZrTe_6_ chain direction that runs along *a* axis (Fig. 4b,c). When we rotated the sample, the quasi-one-dimensional features also rotated simultaneously, indicating it is associated with the intrinsic property of ZrTe_5_ rather than an artefact or a photoemission matrix element effect (Supplementary Fig. 11). To firmly establish the validation of the quasi-one-dimensional feature and its possible origin, we have carried out massive experiments with a plenty of samples. We found that the signal intensity of these extra bands varies among samples but its observation is quite common in samples with one-dimensional thread structures on surface. The quasi-one-dimensional feature appears only when the sample surfaces are cracked (by cycling of cooling and warming) with a bunch of edges; it is invisible in ZrTe_5_ with a smooth mirror-like surface (Fig. 1). They are not present in the band structure calculations of the bulk ZrTe_5_ (Supplementary Fig. 4) either. These results indicate that the extra bands are closely associated with the one-dimensional edges of the line structures on the ZrTe_5_ surface. There are some qualitative agreements between our measurements and the calculations. First, the extra hole-like bands along Γ–*X* direction (Fig. 4e) are quite similar to those shown by the red line in Fig. 3d in ref. 37, which come from the edge of monolayer ZrTe_5_ with prismatic chain termination. Second, the measured bands have comparable Fermi momenta *k*_F_'s and Fermi velocity with those in the calculations. Third, the edge state bands merge into the bulk bands at ∼150 meV binding energy in both our measurements and the calculations. These results lend more support on the edge states of our observation although further effort is needed to have a more quantitative comparison when the structure of the edges in the cracked ZrTe_5_ samples is known. Our observations are also consistent with the recent scanning tunnelling microscope experiments on ZrTe_5_ that reported observation of edge states on ZrTe_5_ surface^49^,^50^. These results indicate that the extra quasi-one-dimensional bands are consistent with the edge state of single-layer ZrTe_5_ (ref. 37). They therefore suggest that ZrTe_5_ is a weak topological insulator that can be viewed as a stack of two-dimensional topological insulator (or quantum spin Hall insulator) with topological edge mode.

We finally examine on possible temperature-induced topological phase transition that is expected in ZrTe_5_ (ref. 37). Band structure calculations^37^ indicate that the topological property of ZrTe_5_ is sensitive to the interlayer coupling between the adjacent ZrTe_5_ layers and bulk ZrTe_5_ is sitting very close to the border between the weak and strong topological insulators. When the interlayer coupling is weak, bulk ZrTe_5_ acts as a three-dimensional weak topological insulator. When the interlayer coupling gets strong, bulk ZrTe_5_ can be transformed into a three-dimensional strong topological insulator like typical Bi_2_Se_3_ and Bi_2_Te_3_ three-dimensional topological insulators^51^,^52^,^53^. In this case, topological surface state is expected to appear, as shown in Supplementary Fig. 4c,e. With decreasing temperature, the lattice constant *b* decreases (Fig. 3j), the gap between the LB band and the UB band decreases (Fig. 2f); these results are consistent with the enhancement of the interlayer coupling as expected^37^.

The topological transition from a weak to a strong three-dimensional insulators asks that the LB and the UB bands at Γ point move close to each other with decreasing temperature, touch and merge together and then open a new gap after the band inversion. Our ARPES results (Fig. 2) indeed show a trend of gap closing with decreasing temperature, but we believe the gap persists over the entire temperature range that we have measured. One might argue that below 65 K, the filling up of the spectral weight at the valley bottom could be due to appearance of a new surface state that emerges in the new gapped region after the original gap is closed and the band inversion is realized. This possibility cannot be fully ruled out. To check on this possibility, we carefully searched for the possible signature of the topological surface state that would be expected inside the gap region near Γ point at ∼2 K. As seen from Supplementary Fig. 9b,e, no signature of the extra surface state can be resolved. Therefore, our results suggest that till the lowest temperature we measured (∼2 K), the gap between the LB band and the UB band near Γ remains open, and no band inversion has been realized in the measured temperature range. The observation of possible edge states in ZrTe_5_ at 15 K also indicates that ZrTe_5_ is a weak topological insulator (Fig. 4). The lattice shrinkage along *b* axis with temperature (Fig. 3j) by 0.3% is not enough for driving the topological phase transition to occur^37^. Our present study indicates a clear trend of such a transition; further enhancement of the interlayer coupling is needed to materialize such a topological phase transition.

In summary, we have carried out comprehensive high-resolution ARPES measurements on ZrTe_5_. We have uncovered direct electronic evidence of a temperature-induced Lifshitz transition in ZrTe_5_. The sample undergoes a *p*-type semimetal to a semiconductor, to an *n*-type-semimetal transition with decreasing temperature. This evolution provides a natural explanation on the long-standing issue of the origin of the resistivity anomaly in ZrTe_5_. It also indicates that ZrTe_5_ with a resistive anomaly at ∼135 K is not a three-dimensional Dirac semimetal. We observed quasi-one-dimensional electronic features that may be associated with the edge states of ZrTe_5_. Such an observation, together with the persistence of bandgap opening, absence of Dirac surface states and no topological phase transition over the entire temperature range we measured, indicates that ZrTe_5_ is a weak topological insulator. With decreasing temperature, there is a tendency of the transition from a weak topological insulator to a strong topological insulator. Further enhancement of the interlayer coupling, either by external high pressure or internal chemical pressure, may facilitate the realization of such a topological phase transition.

## Methods

### Sample preparation

High-quality single-crystal samples of ZrTe_5_ were grown by the chemical vapour transport method with iodine as transport agent. The unique resistivity peak sits at a temperature of ∼135 K (Fig. 1d), in good agreement with the typical values reported in most of the previous literatures^2^,^3^,^4^,^6^,^7^,^8^,^9^,^10^,^41^,^42^,^44^,^45^.

### High-resolution ARPES measurements

Most of ARPES measurements were performed at our new laser-based system equipped with the 6.994 eV vacuum-ultraviolet laser and the time-of-flight electron energy analyser (ARToF 10 K by Scienta Omicron). This latest-generation ARPES system is capable of measuring photoelectrons covering two-dimensional momentum space (*k*_*x*_, *k*_*y*_) simultaneously. Measurements were performed using both s- and p-polarization geometries. For the photon energy-dependent measurements, our tunable-laser ARPES system equipped with a hemispherical analyser DA30L (Scienta-Omicron) was used with the photon energy varying in the range of 5.9 and 7.09 eV. The overall energy resolution was set at 1–5 meV, and the angular resolution was ∼0.1°. All the samples were measured in ultrahigh vacuum with a base pressure better than 5 × 10^−11^ mbar. The samples for temperature-dependent experiments were cleaved *in situ* at either 35 or 255 K and measured at temperatures ranging from 35 to 255 K. Samples measured at 15 or 2 K were cleaved *in situ* at 15 and 2 K, respectively. To avoid the hydrogen contamination on the sample surface at low temperature, during the ARPES measurements, we deliberately stay away from 20–33 K range near the hydrogen-boiling temperature.

### Band structure calculations

The band structure calculations were performed with the projector augmented wave method^54^,^55^ implemented in Vienna *ab initio* simulation package^56^,^57^. The cut-off energy for the plane wave expansion is set to 450 eV. The generalized gradient approximation of Perdew–Burke–Ernzerhof type^58^ is used to deal with the exchange and correlation potential. The *k*-point sampling grid in the self-consistent process is 13 × 13 × 7. Spin Orbital Coupling (SOC) is included as a second vibrational step using scalar relativistic eigenfunctions as the bases after the initial calculation is achieved in the self-consistent iterations. Maximally localized Wannier functions for the *p* orbitals of Te atoms have been constructed by using the WANNIER90 code^59^,^60^,^61^. A slab tight-binding model has been constructed to calculate the surface states by using the Wannier functions.

### Data availability

The authors declare that all data supporting the findings of this study are available within the paper and its Supplementary Information files.

## Additional information

**How to cite this article:** Zhang, Y. *et al*. Electronic evidence of temperature-induced Lifshitz transition and topological nature in ZrTe_5_. *Nat. Commun.* **8**, 15512 doi: 10.1038/ncomms15512 (2017).

**Publisher's note:** Springer Nature remains neutral with regard to jurisdictional claims in published maps and institutional affiliations.

## Supplementary Material

Supplementary Information Supplementary Figures, Supplementary Notes, Supplementary References.
