# Supplementary material for: Electronic evidence of temperature-induced Lifshitz transition and topological nature in ZrTe5
Source: Nat Commun. 2017 May 23;8:15512. doi: 10.1038/ncomms15512 (PMC5457516; doi:10.1038/ncomms15512)
Supplement: Supplementary Information — Supplementary Figures, Supplementary Notes, Supplementary References. [file ncomms15512-s1.pdf]

**Supplementary Note 1. Electronic Structure of $ZrTe_5$ Measured under s- and p-Polarization Geometries**

In order to get full information about the electronic structure of $ZrTe_5$, both **s**- and **p**-polarization geometries were used in our ARPES measurements. In the **s**-polarization geometry, the electric field vector of the incident laser is perpendicular to the plane formed by the incident light and the lens axis of the electron energy analyzer, while in the **p**-polarization geometry, the electric field vector of the incident light lies within such a plane. Supplementary Fig. 1 shows results of the constant energy contours and band structures measured under these two distinct polarization geometries. It is clear that the topology of the constant energy contours and the band dispersions are similar for these two cases. But the spectral weight distribution shows obvious dependence on the polarization geometry, as seen more clearly by comparing high binding energy contours (Supplementary Fig. 1d versus Supplementary Fig. 1j).

**Supplementary Note 2. Detailed Analysis of Bands near Γ Point**

Figure S2 shows band structures of $ZrTe_5$ measured along Γ-Y at two different temperatures (195 K and 2 K) and the corresponding photoemission spectra. The bands near Γ point are rather broad. When taking the second-derivative of the bands with respective to the energy, as usually done in ARPES community to highlight bands (see Supplementary Fig. 2d and Supplementary Fig. 2i), it seems that these lower-branch hole-like bands are composed of two sub-bands, as seen most clearly in Supplementary Fig. 2i. However, a close inspection of the corresponding momentum distribution curves (MDCs) (Supplementary Fig. 2b and Supplementary Fig. 2g) and energy distribution curves (EDCs) (Supplementary Fig. 2c and Supplementary Fig. 2h) indicates that the hole-like energy bands consist of neither one single MDC or EDC peaks, nor two sharp MDC or EDC peaks. This can be seen more clearly in Supplementary Fig. 2e and Supplementary Fig. 2j where the EDCs at the Γ point are plotted. It is clear that, in this case, the seemingly two sub-bands in the second-derivative images (Supplementary Fig. 2e and Supplementary Fig. 2j) do not correspond to two sharp peaks in its corresponding EDCs. Rather, the EDC of the lower-branch hole-like bands represents a broad continuum that is encompassed by two relatively sharp edges. The position of the two "sub-bands" in the second-derivative images actually corresponds to the location of the two edges as marked in Supplementary Fig. 2d-e and Supplementary Fig.

2i-j by pink arrowed lines. These results clearly indicate that the hole-like bands near Γ are not composed of two sub-bands, but represent an envelope of spectral weight encompassed by two edges. The bandwidth of the hole-like band, i.e., the energy difference between the two edges, is maximal at the Γ point, and decreases with the momentum moving away from the Γ point. It is also clear that the bandwidth gets wider with decreasing temperature, as seen from comparing Supplementary Fig. 2(d-e) and Supplementary Fig. 2(i-j).

**Supplementary Note 3. Calculated Electronic Structures of $ZrTe_5$**

Extensive band structure calculations have been performed on $ZrTe_5$ by considering the spin-orbit-coupling effect, surface and bulk states, $k_z$ effect, and the effect of layer distance along $b$-axis on the topological property. Supplementary Fig. 3 shows bulk electronic structure under different layer distance along $b$-axis. It shows a clear topological phase transition from strong topological insulator to weak topological insulator at about $1.04b_0$ ($b_0 = 14.502$ $\mathring{A}$ is the lattice constant $b$ of $ZrTe_5$ at room temperature[1])). Considering the existance of an energy gap and its size at Γ point and the topological nature of $ZrTe_5$ samples, we beileve that our experimental results are more consistent with the calculated results with a layer distance of $1.06b_0$. Supplementary Fig. 4a shows bulk electronic structure of $ZrTe_5$ in the weak topological insulator case. The corresponding constant energy contours at different energies are shown in Supplementary Fig. 5. The primary features of the calculated bulk electronic structure are consistent with ARPES data (Fig. 1) in terms of the evolution of constant energy contours with energy, nearly-linear band dispersions and full gap formation near the Brillouin zone center. However, one apparent discrepancy exists as to the lineshape of the photoemission spectra. The EDC for the lower branch hole-like band is not a single-peak, nor a combination of two sharp peaks, but a spectral continuum encompassed by two edges (Supplementary Fig. 2). In order to understand such a difference, we performed band structure calculations using a slab method. Supplementary Fig. 4b shows the calculated band structure of $ZrTe_5$ using the slab method, by taking the lattice constant along the $b$-axis as $1.06b_0$ to reduce the interlayer interaction that gives rise to a weak three-dimensional topological insulator. Supplementary Fig. 4c shows calculated band structure of $ZrTe_5$ using the slab method by taking the lattice constant along the $b$-axis as $1.0b_0$ which results in a strong three-dimensional topological insulator. In the calculated results using the slab method, the bands are composed of a spectral continuum that are consistent with our measured results.

It is also clear that with the decreasing of the lattice constant $b$, the bandwidth of the LB hole-like band near Γ gets broader; this is also consistent with the measured results (Supplementary Fig. 2) that the bands get broader with decreasing temperature, accompanied by the shrinking of the lattice constant $b$. The broad photoemission spectra in the hole-like bands near Γ can be explained as due to finite $k_z$ resolution in the photoemission process, in conjunction with $k_z$-dependence of the band structure for a three-dimensional electronic structure. In the case of strong topological insulator (Supplementary Fig. 4c), topological surface state appears, as marked by the red lines in Supplementary Fig. 4c and Supplementary Fig. 4e. These surface states were calculated by using the Wannier functions on a slab tight binding model. Its presence can be used as a signature for a strong three-dimensional topological insulator. The evolution of the constant energy contours with energy (Supplementary Fig. 5) for the calculated bulk band structure of $ZrTe_5$ show good agreement with the measured results (Fig. 1e-h) considering the adjustment of the chemical potential between the calculations and experiment. Some qualitative differences as to the bandwidth or band slope between the experiment and calculations are present that may come from $k_z$ effect and other factors. We note that there is some difference of the calculations in Ref.[2] and those in our present paper. The difference comes from different ways of selecting initial parameters for calculations and different software packages used. In Ref.[2], theoretically optimized lattice constants and experimental ones at 10 K, and OpenMX software package, were used. In our present paper, experimental lattice constants at room temperature, and VASP software package, are used. The new calculations show a good agreement with our experimental results.

**Supplementary Note 4. Reproducibility of Temperature-Dependent ARPES Measurements on $ZrTe_5$**

In order to check on the possible sample aging effect and reproducibility of our temperature-dependent ARPES data, we performed experiments at different temperatures by cycling of cooling down and warming up the measured $ZrTe_5$ sample. Supplementary Fig. 6a shows the band structures of $ZrTe_5$, cleaved at 255 K, and measured along Γ-X direction at different temperatures by cooling the sample all the way down to 35 K. After the completion of data acquisition at 35 K, the sample was warmed up gradually to 255 K, and Supplementary Fig. 6b shows the band structures along the same Γ-X direction taken

at different temperatures during warming up the sample. It is clear that the obtained data for a given temperature are highly reproducible during the cooling-down and warming-up processes, indicating that our ARPES measurements are reliable and the sample aging effect is negligible.

**Supplementary Note 5. Large Momentum Space Measurements of $ZrTe_5$ at Different Temperatures**

We have measured the electronic structure of $ZrTe_5$ by covering the full first Brillouin zone at 35 K and 225 K, as shown in Supplementary Fig. 7. We measured several $ZrTe_5$ samples and the results are highly reproducible. At 35 K, in addition to the features near $\Gamma$ (denoted as $\alpha$ hereafter), we observe signature of another set of electron pockets at the Y-M Brillouin zone boundary (denoted as $\beta$ hereafter). This is consistent with the band structure calculations (Supplementary Fig. 5d). Except for these two kinds of electronic structures, no other band crossing the Fermi level is detected over the entire first Brillioun zone in the temperature range we measured.

Supplementary Fig. 8a shows the Fermi surface of $ZrTe_5$ at diferent temperatures covering a large momentum space encompassing both the features at $\Gamma$ and the other $\beta$ feature. The band structure measured along a typical momentum cut (marked as a line in 35 K panel in Supplementary Fig. 8a) at different temperatures are shown in Supplementary Fig. 8b. At low temperature, the observation of four small $\beta$ electron pockets, together with the $\alpha$ electron pocket at $\Gamma$, is consistent with the quantum oscillation measurements where two electron pockets are detected[3, 4]. The appearance of these electron pockets will also enhance electrical conductivity at low temperature.

**Supplementary Note 6. Electronic Structure of $ZrTe_5$ at 2K**

To check whether the gap between the LB and UB bands is closed and possible emergence of the in-gap topological surface state at low temperature, we carried out ARPES measurement on $ZrTe_5$ to a very low temperature, $\sim$2 K (Supplementary Fig. 9). At such an extremely low temperature, we detected no surface state in the bulk gap. It indicates that $ZrTe_5$ is still in weak topological state at 2 K.

**Supplementary Note 7. Electronic Structure of $ZrTe_5$ Measured at Different Photon Energies**

We have carried out ARPES measurements on $ZrTe_5$ samples using various laser photon energies, as shown in Supplementary Fig. 10a. It shows that the gap at $\Gamma$ point decreases with the photon energy changing from 6.13 to 6.99 eV. Our results indicate that the electronic structure measured at 6.99 eV is close to $\Gamma$ point. By taking the inner potential $V_0$ at 7.5eV for $ZrTe_5$ from literature[5]) , we also estimated the $k_z$ for various photon energies, as shown in Supplementary Fig. 10b. The measured data are consistent with the calculation (Supplementary Fig. 4a), indicating that our 6.99eV data is close to the $\Gamma$ point. According to the discussion in the main manuscript, because the relative energy position change of the upper conduction band and lower valence band near $\Gamma$ with temperature, we conclude that the gap exists at $\Gamma$ in the three-dimensional Brillouin zone. The nice agreement between our calculations and measurements shown here is a good indication on the reliability of our band structure calculations.

**Supplementary Note 8. Orientation of Quasi-One-Dimensional Features in $ZrTe_5$**

We measured two samples to check the orientation of the quasi-one-dimensional feature in $ZrTe_5$, as shown in Supplementary Fig. 11. For the sample a, when the cracked lines on the surface are horizontal (Supplementary Fig. 11a), the observed quasi-one-dimensional features run vertically (Supplementary Fig. 11b and Supplementary Fig. 11c). For another sample b, where we put the cracked lines on the surface vertically (Supplementary Fig. 11d), i.e., it is 90 degree rotated with respect to sample a during the measurement, the observed quasi-one-dimensional features run horizontally. This confirms that the quasi-one-dimensional feature is related to the intrinsic property of $ZrTe_5$, rather than an artifact or due to photoemission matrix element effects. It also shows that the observed quasi-one-dimensional feature is closely related to the one-dimensional lines and edges on the sample surface.


[1] Okada, S. et al. Giant resistivity anomaly in $ZrTe_5$. J. Phys. Soc. Jpn. 49, 839 (1980).

[2] Wen, H. M. et al. Transition-metal pentatelluride $ZrTe_5$ and $HfTe_5$: A paradigm for large-gap quantum spin Hall insulators. Phys. Rev. X 4, 011002 (2014).

[3] Kamm, G. N. et al. Fermi surface, effective masses, and Dingle temperatures of $ZrTe_5$ as derived from the Shubnikov–de Haas effect. Phys. Rev. B 31, 7617 (1985).

[4] Mitsuru, I. et al. Shubnikov-de Haas oscillations and Fermi surfaces in transition-metal pentatellurides $ZrTe_5$, and $HfTe_5$. J. Phys. C: Solid State Phys. 20, 3691-3705 (1987).

[5] Moreschini, L. et al. Nature and topology of the low-energy states in $ZrTe_5$. Phys. Rev. B 94, 081101(R) (2016).

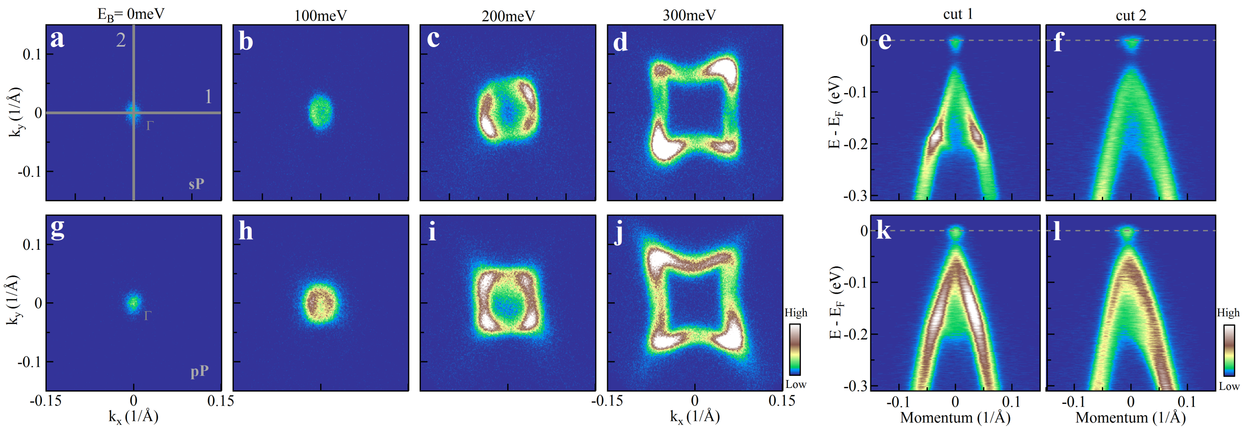


Supplementary Figure 1: **Electronic structure of $ZrTe_5$ measured at 35 K under two different polarization geometries**. (a-d) Constant energy contours of $ZrTe_5$ at different binding energies of 0 (a), 100 meV (b), 200 meV (c) and 300 meV (d) measured under *s*-polarization geometry. The spectral weight intensity is obtained by integrating within 10 meV energy window with respect to the respective binding energy. (e-f) Corresponding band structures along cut 1 (Γ-X direction) and cut 2 (Γ-Y direction). The location of cut 1 and cut 2 is indicated in (a). (g-j) and (k-l) are constant energy contours and corresponding band structures of $ZrTe_5$ measured under *p*-polarization geometry.

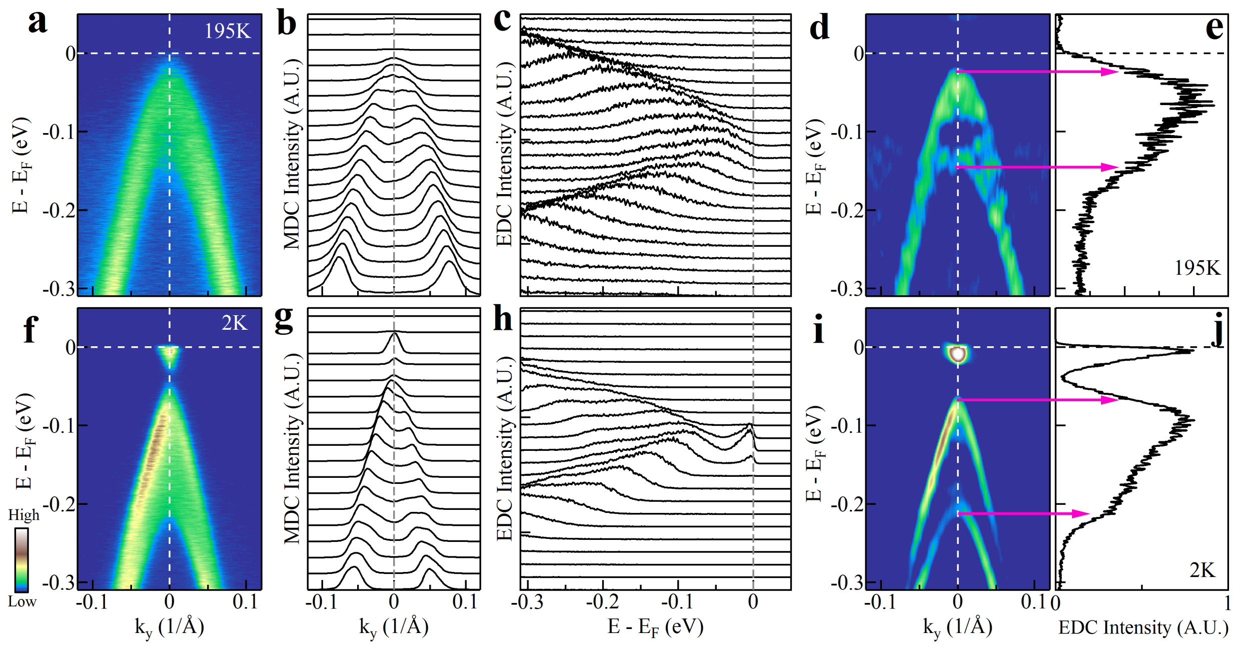


Supplementary Figure 2: **Linesahpe analysis of $ZrTe_5$ bands near Γ point** . (a) Band structure of $ZrTe_5$ measured at 195 K along Γ-Y direction (cut 3 in Fig. 1g). Its corresponding MDCs at different binding energies are shown in (b), and corresponding EDCs at different momenta are shown in (c). (d) Second derivative image with respect to energy for (a). (e) EDC at Γ point in (a). (f) Band structure of $ZrTe_5$ measured at 2 K along Γ-Y direction (cut 3 in Fig. 1g). Its corresponding MDCs at different binding energies are shown in (g), and corresponding EDCs at different momenta are shown in (h). (i) Second derivative image with respect to energy for (f). (j) EDC at Γ point in (f).

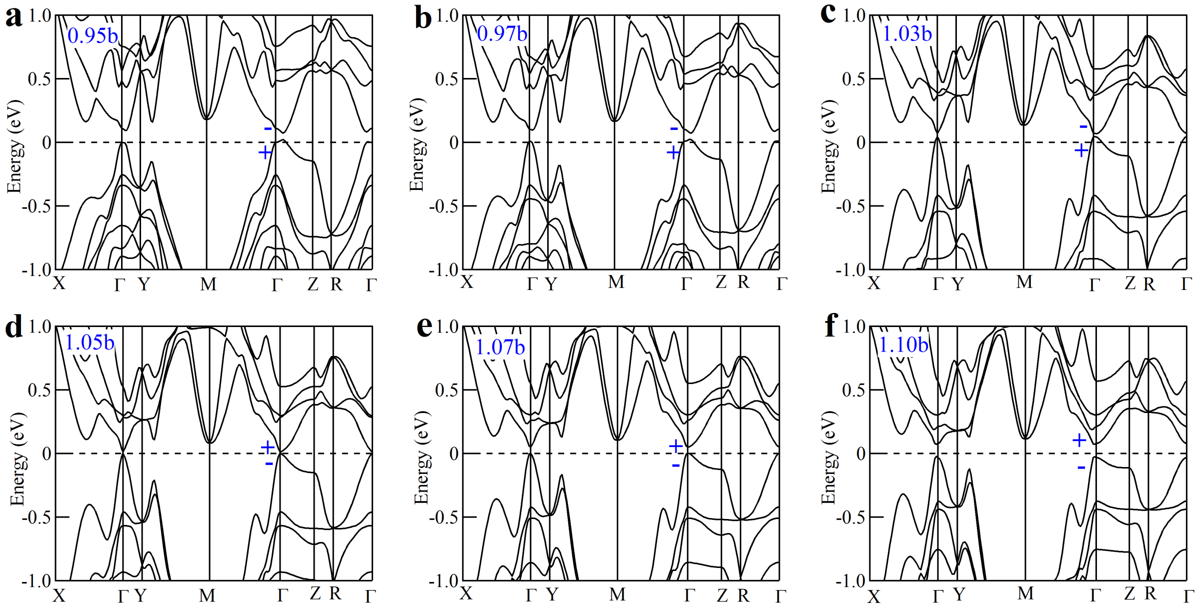


Supplementary Figure 3: **Calculated bulk band structure of $ZrTe_5$ with different lattice constant *b*.** (a-c) bulk band structure in strong topological insulator case with band inverse. (d-f) bulk band structure in weak topological insulator case.

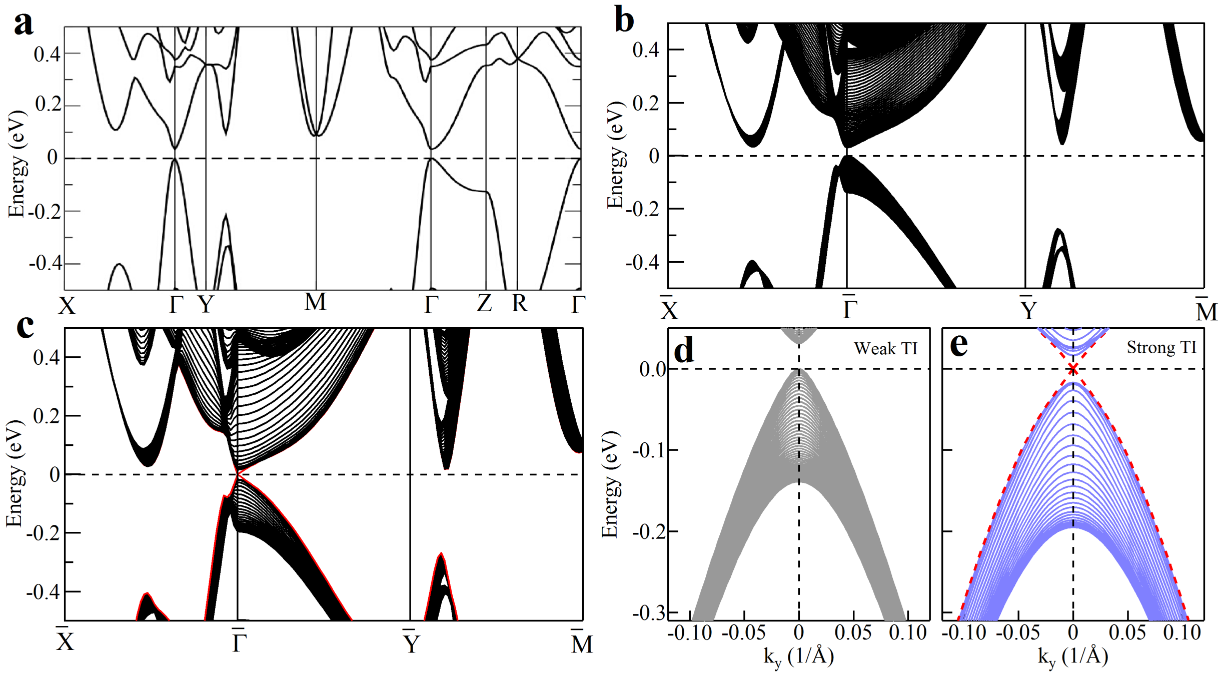


Supplementary Figure 4: **Calculated band structures of $ZrTe_5$.** (a) Calculated bulk band structure. (b) Calculated band structure by slab method, under the condition of weak topological insulator. The lattice constant along the $b$-axis is taken as $1.06b_0$ with $b_0$=14.502$\AA$ being the lattice constant $b$ of $ZrTe_5$ at room temperature[1] . (c) Calculated band structure by slab method under the condition of strong topological insulator. The lattice constant $b$ is taken as $1.0b_0$. Red lines represent topological surface state. (d) Expanded view of (b) for the calculated band structure around Γ point along Γ-Y direction for the weak topological insulator case. (e) Expanded view of (c) for the calculated band structure around Γ point along Γ-Y direction for the strong topological insulator case. The red dashed lines represent topological surface state emerged in the gapped region.

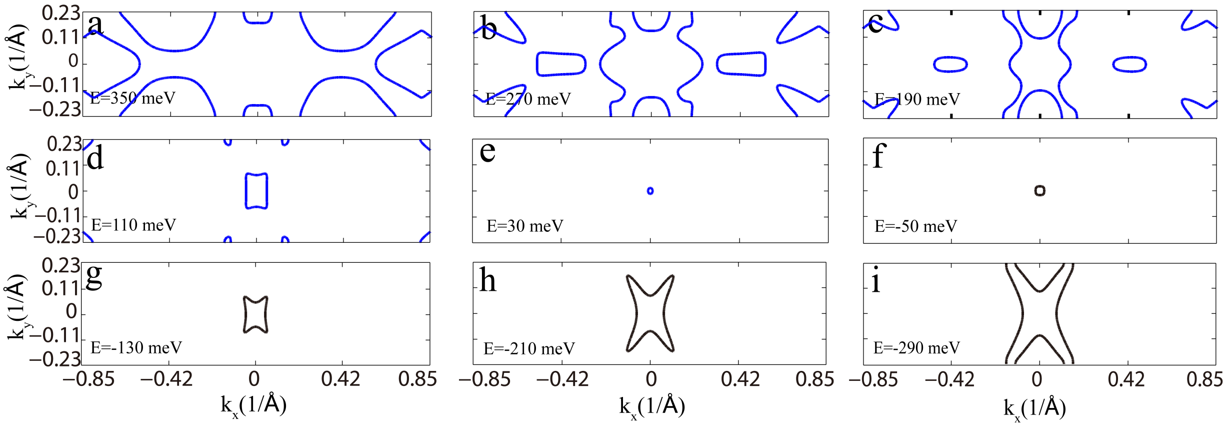


Supplementary Figure 5: **Constant energy contours from bulk band structure calculations of $ZrTe_5$ with $k_z$=0.2 $\pi/b$.** (a-e) Constant energy contours at different energies above the Fermi level. At the energy of 110 meV (d), there is an electron pocket at Γ, another set of electron pockets near (±0.14,±0.22) $1/\AA$, and the third set of electron pockets near M. At the energy of 30 meV (e), only a tiny electron pocket exists near Γ. (f-i) show constant energy contours below the Fermi level. In this case, only the hole-like pockets are present near Γ. It increases in size with increasing binding energy and becomes a warped rectangle at high binding energies like 130 meV (g), 210 meV (h) and 290 meV (i).

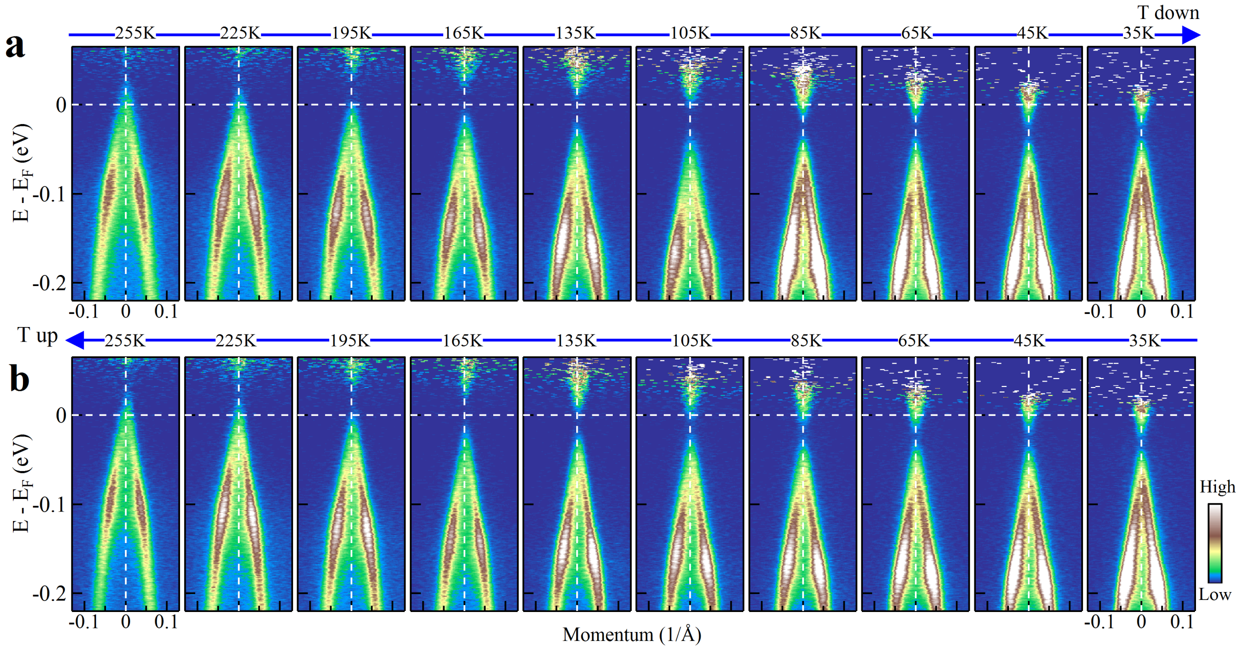


Supplementary Figure 6: **Reproducibility of temperature-dependent ARPES measurements on $ZrTe_5$.** (a) Band structures of $ZrTe_5$ measured along the Γ-X direction (cut 1 in Fig. 1g) at different temperatures. The sample was cleaved at 255 K and measured at different temperatures during cooling down. (b). Band structures at different temperatures when the sample was warming up. It is clear that the temperature-dependent data are highly reproducible when measured during the cooling down and warming up processes.

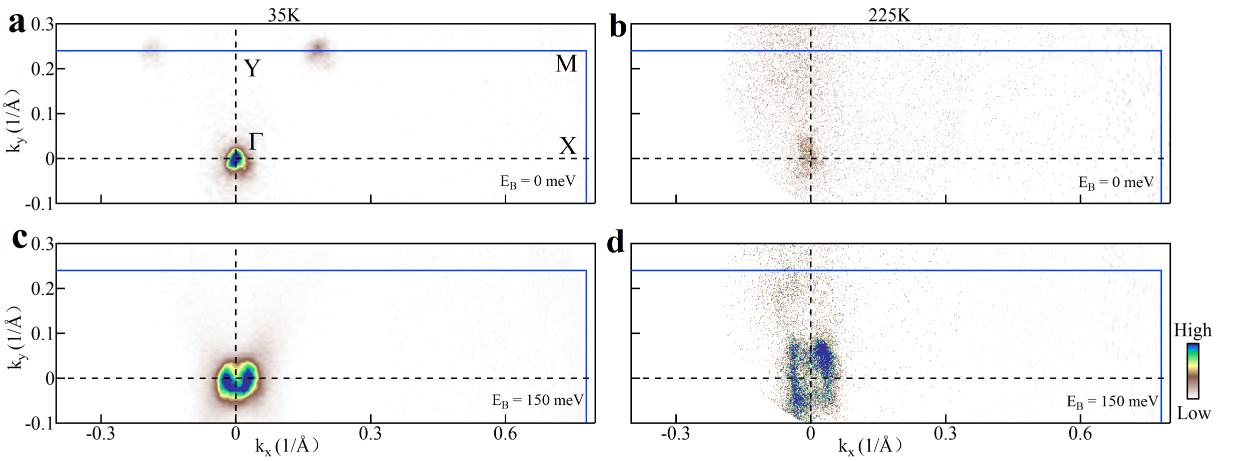


Supplementary Figure 7: **Constant energy contours of $ZrTe_5$ covering the full first Brillouin zone.** (a-b) Fermi surface of $ZrTe_5$ measured at 35 K and 225 K, respectively. (c-d) Constant energy contours at 150 meV binding energy measured at 35K and 225K, respectively.

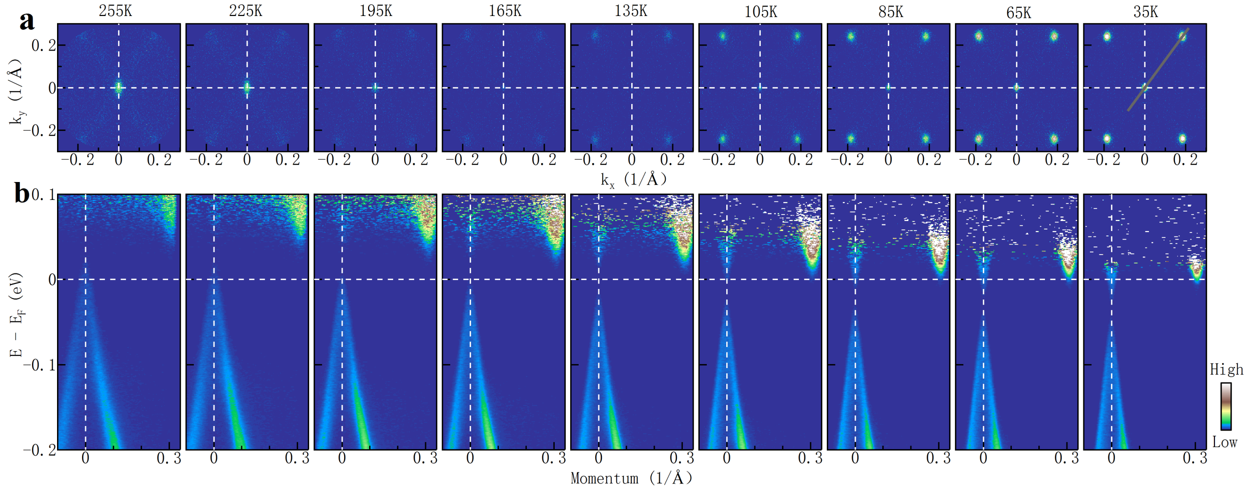


Supplementary Figure 8: **Temperature-dependent electronic structure of $ZrTe_5$ covering a large momentum space.** (a) Fermi surface of $ZrTe_5$ measured at different temperatures covering both the $\alpha$ pocket at Γ and $\beta$ feature along the YM edges. (b) Band structure of $ZrTe_5$ measured at different temperatures along a typical momentum cut, as marked by a line in 35 K panel in (a), that crosses both the $\alpha$ and $\beta$ features.

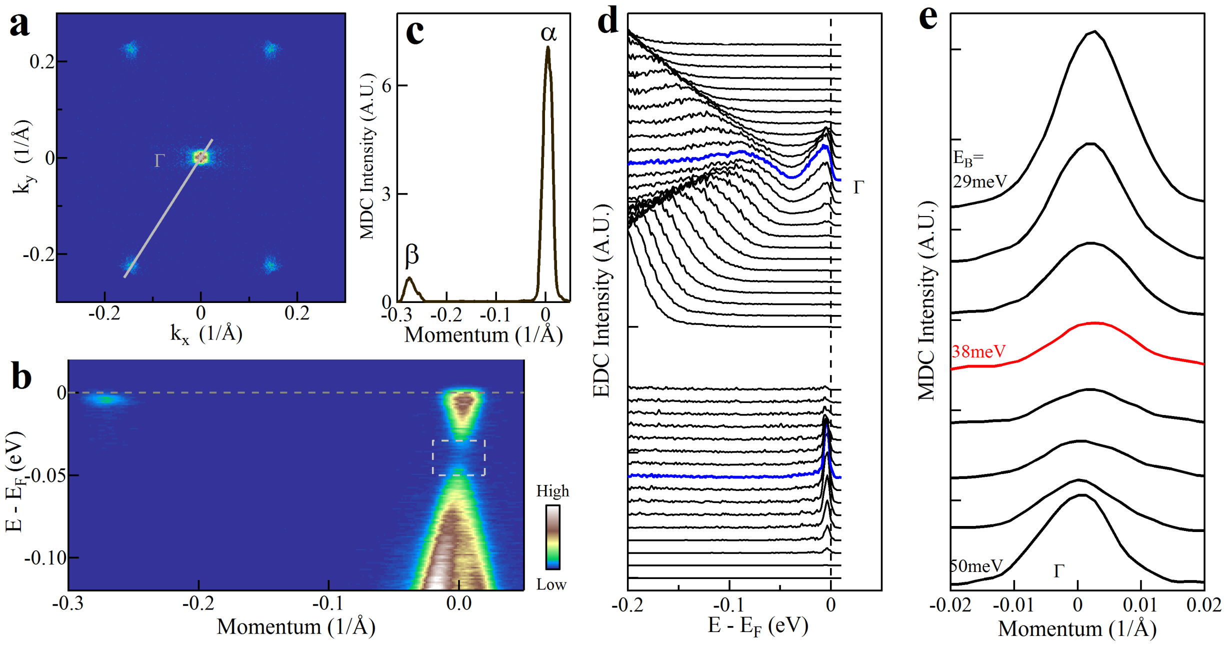


Supplementary Figure 9: **Electronic structure of $ZrTe_5$ measured at a lower temperature of ∼2 K.** (a) Measured Fermi surface around the Brillouin zone center including an electron pocket at Γ and four $\beta$ electron pockets near the zone edge. (b) A typical band structure measured along the momentum cut marked in (a) that crosses two electron pockets. (c) Corresponding MDC for the band structure in (b) at the Fermi level showing two peak structures. (d) Photoemission spectra (EDCs) near these two electron pockets. The EDCs at Γ and at the center of the $\beta$ pocket are marked as blue lines. There are sharp EDC peaks for the corner $\beta$ pocket. The observed band is very shallow; the band bottom is only ∼5 meV below the Fermi level. (e) Momentum distribution curves near the gapped region marked by the dashed square in (b) between the binding energy of 50 meV and 29 meV. The MDC at the gap center at a binding energy of 38 meV is marked as red.

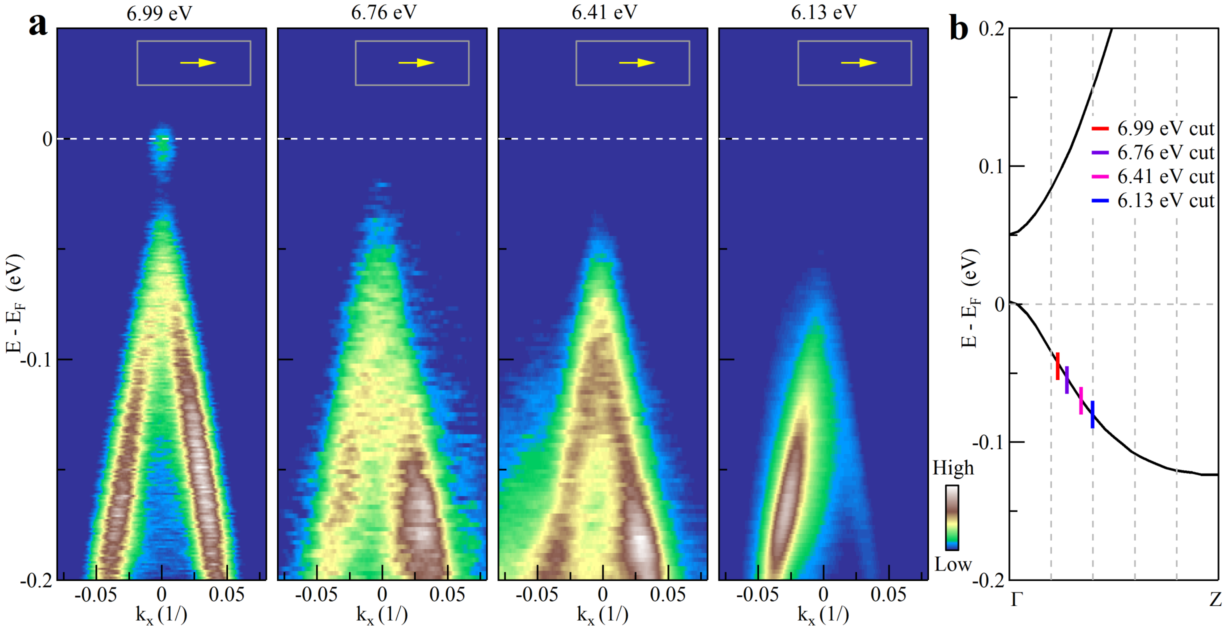


Supplementary Figure 10: **Band structure of $ZrTe_5$ meaured at 35 K along Γ-X direction using different photon energies.** (a) Band structure of $ZrTe_5$ measured alomg Γ-X direction at 6.99 eV, 6.76 eV, 6.41 eV and 6.13 eV photon energies. These bands are all measured under *p*-polarization geometry. (b) Calculated bulk band structure of $ZrTe_5$ along Γ-Z for a weak topological insulator case. The estimated $k_z$ position of each laser photon energy in (a) is marked by different color bars.

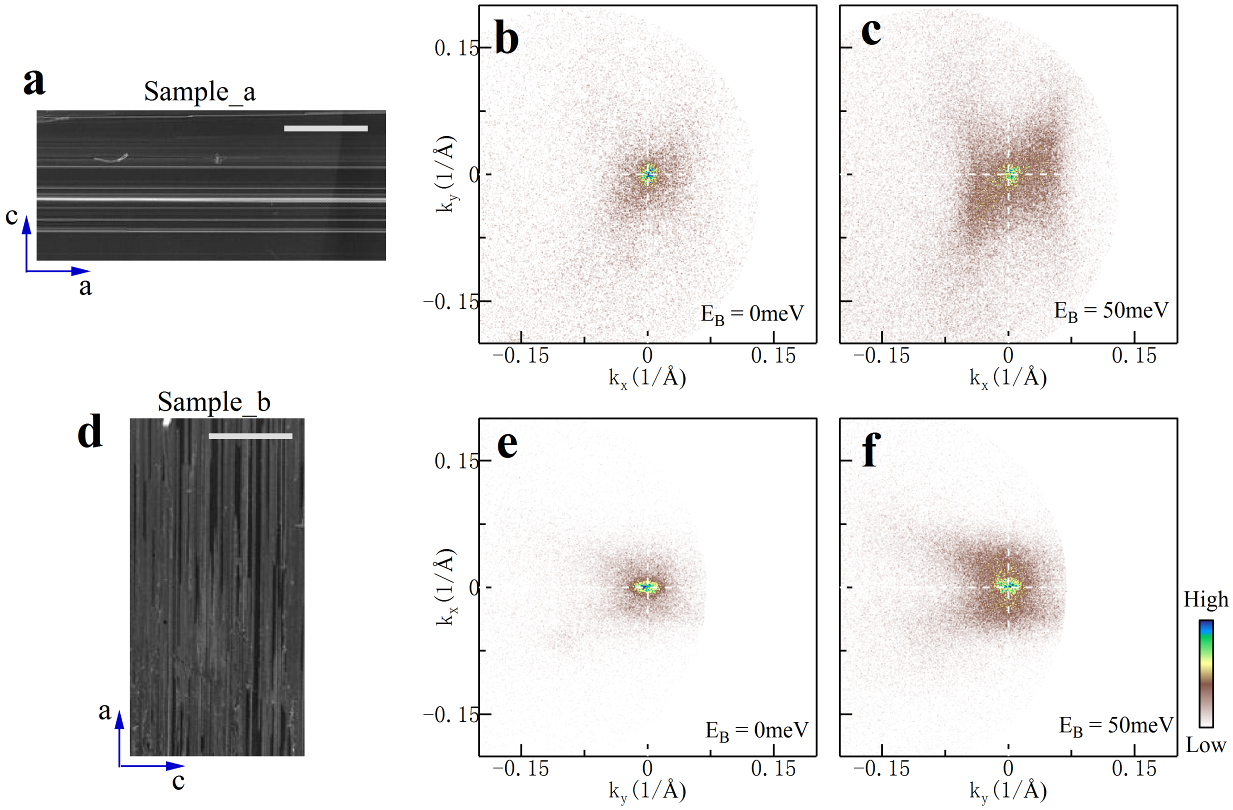


Supplementary Figure 11: **Orientation of quasi-one-diemsnioanl features in two cracked $\mathbf{ZrTe_5}$ samples.** (a) Scanning electron micrsocope (SEM) image of a cleaved $ZrTe_5$ sample *a* surface. The lines and edges run horizontally in this case. (b-c) Constant energy contours at binding energies of 0 (b) and 50 meV (c) for the sample *a*. (d) SEM picture of another cleaved $ZrTe_5$ sample *b* surface. In this case, the lines and edges on the surface were aligned 90 degree rotated from the sample *a* during the measurement. (e-f) Constant energy contours at binding energies of 0 (e) and 50 meV (f) of sample *b*. The scale bar in panel a and d represent 300 um.
